# Psychological Resilience Among Older Japanese Adults With Mild Cognitive Impairment During the COVID-19 Pandemic

**DOI:** 10.3389/fpsyt.2022.898990

**Published:** 2022-06-10

**Authors:** Nanae Matsumoto, Taiki Sugimoto, Yujiro Kuroda, Kazuaki Uchida, Yoshinobu Kishino, Hidenori Arai, Takashi Sakurai

**Affiliations:** ^1^Center for Comprehensive Care and Research on Memory Disorders, National Center for Geriatrics and Gerontology, Obu, Japan; ^2^Department of Prevention and Care Science, Research Institute, National Center for Geriatrics and Gerontology, Obu, Japan; ^3^Department of Public Health, Graduate School of Health Sciences, Kobe University, Kobe, Japan; ^4^Department of Cognitive and Behavioral Science, Graduate School of Medicine, Nagoya University, Nagoya, Japan; ^5^National Center for Geriatrics and Gerontology, Obu, Japan

**Keywords:** older adults, mild cognitive impairment, psychological resilience, CD-RISC-10, COVID-19, sleep quality, depressive symptoms

## Abstract

Psychological resilience refers to the ability to cope with adversities, and deficits in resilience might lead to mental illness. The COVID-19 pandemic has had impact on psychological resilience for older adults, but there are as yet no data on its impacts on the mental health of older adults who were living with mild cognitive impairment (MCI). Therefore, the aim of this study was to investigate the impact of the COVID-19 pandemic on psychological resilience in older adults with MCI and to explore associated physical and psychosocial factors. In this cross-sectional study of 268 older adults aged 65–85, we defined MCI as age- and education-adjusted cognitive decline with a standard deviation of 1.0 or more from the reference threshold. During December 2020 to April 2021, we carried out to all participants the 10-item version of the Connor-Davidson Resilience Scale (CD-RISC-10) to measure psychological resilience. We also conducted a comprehensive geriatric assessment including sleep quality and depressive symptoms (Pittsburgh Sleep Quality Index and 15-item Geriatric Depression Scale, respectively). To identify factors associated with CD-RISC-10 scores (mean: 23.3 ± 0.4), multiple regression analysis revealed that older age [coefficient = 0.23, 95% confidence interval (CI) = 0.06–0.39] was significantly correlated with higher scores, whereas poor sleep quality (coefficient = −2.06, 95% *CI* = −3.93 to −0.19) and depressive symptoms (coefficient = −2.95, 95% *CI* = −5.70 to −0.21) were significantly correlated with lower scores. In this study, older adults with MCI showed low psychological resilience during the COVID-19 pandemic, and people with low psychological resilience indicated poor sleep quality and depressive symptoms. Our findings suggest directions for devising interventions to maintain mental health and psychological resilience among the vulnerable population of older adults with MCI living under the socially isolated conditions of COVID-19 pandemic restrictions. Our recommendation includes continuous assessment of this population and appropriate care for poor sleep quality and depressive symptoms.

## Introduction

The coronavirus disease 2019 (COVID-19) pandemic caused by severe acute respiratory syndrome coronavirus 2 (SARS-CoV-2) began spreading in Japan in January 2020. Prevention measures such as social distancing effectively reduced new infections (1), but these measures severely restricted older adults' participation in physical, social, and community activities (2, 3). Although the number of participants was limited, the agitation, depression and anxiety of older adults with mild cognitive impairment (MCI) increased during lockdown (4). MCI or subjective cognitive decline showed decreased physical activity (43.4%) since the start of the lockdown, and there was an increase of 69.6% in the time spent sitting or lying down (5). These reports suggest that older adults with MCI have faced problems in mental health and physical activity during the COVID-19 pandemic.

Researchers found that isolation caused by infection prevention measures was linked to depression, anxiety, and cognitive decline as well as lower self-worth, which are factors involved in the concept of resilience (6).

Resilience is the ability to adapt positively in the face of adversity and maintain mental health (7), and it derives from a combination of genetic, biological, psychological, social, and cultural factors (8, 9). In one study before COVID-19, older adults showed higher psychological resilience than did young adults (10), although older adults with MCI showed lower resilience than did healthy older adults (11). High resilience is useful for recovering from stress (12), but some researchers found lower psychological resilience among adults during the pandemic compared with before (13–16). Given that older adults with MCI frequently showed mental health problems during the pandemic such as fear, anxiety, and frustration (17, 18), it is understandable that their psychological resilience would be affected as well. However, no researchers have yet investigated psychological resilience in older adults with MCI during the COVID-19 pandemic including factors related to their resilience. Therefore, we aimed with the present study to investigate these factors and the psychological resilience of older adults with MCI during the COVID-19 pandemic. Our identifying psychological resilience and these associated factors should help with developing effective mental health interventions for older adults with MCI.

## Methods

### Study Design

We conducted this cross-sectional study as part of the World-Wide FINGERS SARS-CoV-2 survey (19) in World-Wide FINGERS (20), the global network of lifestyle intervention trials for dementia risk reduction and prevention. The 10-item version of the Connor-Davidson Resilience Scale (CD-RISC-10) (21) was used in the survey, which was conducted between December 2020 and April 2021, and a comprehensive geriatric assessment (CGA) in the present study was conducted between February 2020 and March 2021.

### Participants

We recruited all participants from the Japan-multimodal intervention trial for prevention of dementia (J-MINT) conducted by the National Center for Geriatrics and Gerontology (NCGG) in Aichi Prefecture (22). The inclusion criterion of this study was older adults with MCI in the age group of 65–85 years. The diagnosis of MCI was made using the NCGG Functional Assessment Tool (FAT), which has been established as a screening tool for older adults at high risk of incident dementia (23, 24). All participants had age- and education-adjusted cognitive decline with a standard deviation (SD) of 1.0 or more from the reference threshold on at least one of the four cognitive domains of memory, attention, executive function, and processing speed as measured by the NCGG-FAT. The exclusion criteria were older adults diagnosed with dementia and having a Mini-Mental State Examination (MMSE) (25) score of < 24 points at baseline; respondents who self-reported a diagnosis of depression and those who had missing data on the CD-RISC-10 were excluded. Of 361 J-MINT participants at the NCGG, 298 took part in the present study. This study was approved by the NCGG Ethics Committee, and all participants underwent informed consent procedures prior to enrolling in the study, all participants gave their consent for participation in the study.

### Measurements

#### Demographic Characteristics

We collected participants' demographic information (age, sex, marital status, living status, years of education, employment status, household income, absolute alcohol consumption per day, smoking status, polypharmacy, and self-reported medical history) by questionnaire. The response options for household income were in increments of JPY 2,000,000, and for self-reported medical history, we asked about the following diseases: diabetes, hypertension, dyslipidemia, atrial fibrillation, congestive heart failure, chronic kidney disease, liver disease, cerebrovascular disease, malignant tumor, thyroid disease, coronary artery disease, neurodegenerative disease, depression and insomnia. For our analyses, the self-reported medical history was divided no medical condition or one or more.

#### CD-RISC-10

We used the CD-RISC-10 score to evaluate respondents' psychological resilience. Respondents rate each item on a scale from 0 (not true at all) to 4 (true nearly all the time), so that the total score ranges from 0 to 40. Higher scores reflect greater psychological resilience.

#### Comprehensive Geriatric Assessment

To explore factors related to the older adults' psychological resilience in this study, we conducted a CGA, a inclusive method of assessing psychological and functional capability of older adults (26). For all participants, the CGA consisted of measuring physical performance, lifestyle, social participation, mental health, and cognitive function with the following full test battery: (1) We used the frailty phenotype proposed by Fried et al. (27), in the Cardiovascular Health Study to measure physical frailty (not frail, prefrail, or frail). (2) We used the Barthel Index (28) to assess basic activities of daily living (ADLs); this scale ranges from 0 to 100, with 100 points indicating complete independence. (3) We measured instrumental ADLs using the Lawton Index, for which perfect scores are 5 for men and 8 for women (29). (4) We used the EuroQol 5-Dimension (EQ−5D) to measure health-related quality of life. The scores for the five EQ-5D dimensions are combined to obtain up to 3,125 possible health states, from which a signal index (utility) score is computed; one score indicates better health status (30). (5) We measured the older adults' nutritional status with the Mini-Nutritional Assessment Short-Form (MNA-SF) (31), which consists of six items (score range: 0–14 points, normal ≥ 12). (6) Sleep quality was evaluated by the Pittsburgh Sleep Quality Index (PSQI); the score ranges from 0 to 21, and a score of 6 or higher indicates poor sleep quality (32). (7) We used the Lubben Social Network Scale-6 (LSNS-6) to measure participants' social networks and connections (33); the LSNS-6 consists of six items, the score ranges from 0 to 30, and scores of 11 or lower indicate social isolation. (8) We measured social participation by asking participants if they belonged to any of eight types of organizations presented in a list (34). (9) We based global cognitive functioning on the MMSE scores, which ranged from 0 to 30. (10) We conducted the 15-item Geriatric Depression Scale (GDS) to measure depression; the score ranges from 0 to 15, and higher scores indicate depressive symptoms (35). For the Japanese version of the GDS, 7 or more points indicates depressive symptoms (36).

### Statistical Analysis

All participants' demographic information is expressed as mean ± SD, median and interquartile range (IQR) or number of people and percentage. We used simple regression to analyze the associations between the CD-RISC-10 and each CGA variable, and we used multiple regression to analyze the CD-RISC-10-variable relationships that were statistically significant in the simple regressions, with the CD-RISC-10 score as the response variable and the statistically significant variables as explanatory variables. Moreover, we entered sex and education, which were related to psychological resilience in a previous study (16), as confounding variables. We conducted all analyses in Stata 16.1 (Stata Corp, College Station, TX, United States) and set *P* < 0.05 as statistically significant.

## Results

Of 298 original participants, 279 responded to the questionnaire of the CD-RISC-10 between December 2020 and April 2021 (response rate: 93.6%). From those 279, we excluded six respondents who self-reported depression and five whose CD-RISC-10 responses were incomplete, which left the data on 268 participants for the analysis. Table 1 shows the demographic characteristics of the participants in this study. The mean CD-RISC-10 score was 23.3 points.

**Table 1:** Demographic characteristics and comprehensive geriatric assessment results.

	**All participants (*n* = 268)**
**Attribute information**	
Age, mean ± SD	74.2 (5.0)
Female, *n* (%)	107 (39.9)
Marital status: married, *n* (%)	208 (77.6)
Living status: with someone, *n* (%)	242 (90.3)
Education, mean ± SD	12.5 (2.4)
Employment status: paid or self-employed, *n* (%)	59 (22.0)
Household income: *n* (%)	
< JPY 2,000,000	37 (13.8)
JPY 2,000,000–3,990,000	145 (54.1)
JPY 4,000,000–5,990,000	44 (16.4)
JPY 6,000,000–7,990,000	21 (7.8)
JPY 8,000,000–9,990,000	16 (6.0)
JPY 10,000,000–and above	5 (1.9)
Absolute alcohol, g/day, mean ± SD	8.6 (15.9)
Current smoker, *n* (%)	17 (6.3)
Polypharmacy, *n* (%)	80 (29.2)
One or more medical conditions, *n* (%)	211 (78.7)
**Physical performance and lifestyle**	
Physical frailty: *n* (%)	
Not frail	113 (50.9)
Prefrail	97 (43.7)
Frail	12 (5.4)
Barthel Index, median (IQR)	100 (100, 100)
Lawton Index	
Women, median (IQR)	8 (8, 8)
Men, median (IQR)	5 (5, 5)
EQ-5D, mean ± SD	0.9 (0.1)
MNA-SF, median (IQR)	13 (12, 14)
PSQI: poor sleep quality, *n* (%)	74 (27.6)
**Social participation, cognitive function, and mental health**	
LSNS-6: socially isolated, *n* (%)	74 (27.6)
Social participation, mean ± SD	1.4 (1.3)
MMSE, median (IQR)	28 (26, 29)
CD-RISC-10, mean ± SD	23.3 (0.4)
GDS: depressive symptoms, *n* (%)	29 (10.8)

*CD-RISC-10, 10-item Connor-Davidson Resilience Scale; EQ-5D, EuroQol-5Dimensions; GDS-15, 15-item Geriatric Depression Scale; LSNS-6, Lubben Social Network Scale-6; MMSE, Mini-Mental State Examination; MNA-SF, Mini-Nutritional Assessment Short-Form; PSQI, Pittsburgh Sleep Quality Index*.

Simple regression analysis showed that higher CD-RISC-10 score was significantly associated with older age and higher household income and lower score was related to social isolation, depressive symptoms, and poor sleep quality (Table 2). The CD-RISC-10 score was not associated with sex, marital status, living status, years of education, employment status, absolute alcohol consumption per day, smoking status, polypharmacy, or one or more medical conditions. In the multiple regression analysis of all statistically significant variables from the simple regressions, older age (coefficient = 0.23, 95% *CI* = 0.06–0.39) was related to higher CD-RISC-10 score, and depressive symptoms (coefficient = −2.95, 95% *CI* = −5.70 to −0.21) and poor sleep quality (coefficient = −2.06, 95% *CI* = −3.93 to −0.19) were associated with lower score (Table 2). The CD-RISC-10 score was not associated with sex, years of education, household income, or LSNS-6 scores.

**Table 2:** Simple and multiple regression analysis results for psychological resilience.

	**Simple regression analysis**		**Multiple regression analysis**	
	**Coefficient**	**95% CI**	**Coefficient**	**95% CI**
Age	0.22	0.05 to 0.39^*^	0.23	0.06 to 0.39^**^
Sex: female	−1.22	−2.95 to 0.52	−1.14	−2.91 to 0.62
Marital status: married	−0.01	−2.05 to 2.04	–	–
Living status: with someone	0.11	−2.77 to 2.99	–	–
Education	0.09	−0.27 to 0.45	0.02	−0.35 to 0.39
Employment status: paid or self-employed	1.58	−0.47 to 3.63		
Household income	0.12	−2.07 to 2.31^*^	0.53	−0.23 to 1.30
Absolute alcohol	0.04	−0.01 to 0.09	–	–
Smoking: current smoker	0.53	−2.97 to 4.02	–	–
Polypharmacy	1.03	−0.84 to 2.91	–	–
One or more medical condition	1.46	−0.61 to 3.54	–	–
Physical frailty				
Prefrail (vs. not frail)	0.27	−1.63 to 2.18	–	–
Frail (vs. not frail)	−0.12	−4.29 to 4.06	–	–
Barthel Index	−0.08	−0.36 to 0.20	–	–
Lawton Index	−0.39	−0.95 to 0.16	–	–
EQ−5D	4.81	−3.72 to 13.3	–	–
MNA-SF	−0.12	−0.64 to 0.40	–	–
PSQI: poor sleep quality	−2.84	−4.71 to −0.97^**^	−2.06	−3.93 to −0.19^*^
LSNS-6: social isolation	−2.12	−4.00 to −0.23^*^	−1.72	−3.67 to 0.22
Social participation	0.40	−0.24 to 1.05	–	–
MMSE	−0.43	−0.90 to 0.04	–	–
GDS: depressive symptoms	−4.04	−6.74 to −1.34^**^	−2.95	−5.70 to −0.21^*^

* *P < 0.05*.

** *p < 0.01*.

*In multiple regression analysis, sex and education were entered as confounding variables. CD-RISC-10, 10-item Connor-Davidson Resilience Scale; EQ-5D, EuroQol-5Dimensions; GDS-15, 15-item Geriatric Depression Scale; LSNS-6, Lubben Social Network Scale-6; MMSE, Mini-Mental State Examination; MNA-SF, Mini-Nutritional Assessment Short-Form; PSQI, Pittsburgh Sleep Quality Index*.

## Discussion

Older adults with MCI had frequent mental health problems during the COVID-19 pandemic (17, 18), psychological resilience is also possibility to be affected, but no previous researchers have investigated the psychological resilience during COVID-19 of older adults who were living with MCI. With the present study, therefore, we clarified psychological resilience in this population, and we identified a number of relevant correlations as below.

The older adults in this study showed a mean CD-RISC-10 score of 23.3 points, which contrasted with scores of 31.7 and 31.1 points in, respectively, adults age 18 or older and older adults who had good cognitive function (16, 37). Researchers who measured resilience with a different test from the CD-RISC-10 found low resilience among older adults with cognitive impairment (11). Moreover, in a previous study during the COVID-19 pandemic, the mean CD-RISC-10 score for older adults was 28.4 points (14). Although we cannot directly compare the mean CD-RISC-10 score from the present study with the scores from previous studies, our findings do suggest lower psychological resilience among older adults with MCI during the pandemic; the mean score in the present study was similar to the mean of 23.6 points that other researchers found for veterans with post-traumatic stress disorder and depression (38). Owing to the nature of cross-sectional studies, we could not describe the pandemic's specific impacts on psychological resilience. However, given that resilience indicates stress coping ability (15, 16), our findings suggest that the major stress from the COVID-19 infection prevention and control measures in Japan put older adults with MCI at high risk for adverse health outcomes.

Many previous researchers reported that psychological resilience was related to age, sex, education level, financial situation, sleep quality, and depressive symptoms (10, 16, 37, 39–41), but we did not find associations with sex or education level. Researchers have reported conflicting results of higher resilience among men, higher resilience among women, and no gender differences (42–44), and other scholars found that higher levels of education were related to higher resilience (16). In our study, participants had a mean education level of 12.5 ± 2.4 years, and the group differences were small, which is likely why we did not find the association between education level and psychological resilience. We also found in the present study an association between high CD-RISC-10 score and older age, which supported earlier findings from reports on psychological resilience and age of higher psychological resilience among older adults than among adults who were middle-aged and younger (10, 39).

Some investigators found significant associations between good sleep quality and high resilience among adults aged 60 years or younger (40, 41), and we also found this significant association. Researchers identified poor sleep quality in respondents with a mean age of 35 during the COVID-19 pandemic (45), and there was another report of increased sleep latency, a component of sleep quality, during the pandemic compared with before (46). It was suggested that people with low psychological resilience are at risk of poor quality during the pandemic.

We also found in the present study a correlation between lower psychological resilience and depressive symptoms, which supported Gerino et al.'s (47), reporting that high resilience contributed to less anxiety and depression. Some people who experience prolonged stress develop psychiatric disorders such as depression, whereas many people can maintain normal psychological functioning through stress, and resilience might be a factor in this normal functioning (48). Researchers found higher prevalence of depressive symptoms among adults during COVID-19 than before (49), and in our study, older adults with depressive symptoms also had low psychological resilience, which would interfere with their coping capacities.

In simple regression analysis in this study, higher household income was related to higher psychological resilience, and social isolation was related to lower resilience. Higher incomes allow for more comfortable and secure lives (50), social ties play a beneficial role in maintaining psychological wellbeing (51), those could be related to higher resilience.

There were some limitations in the present study. First, we collected the data of the CD-RISC-10 for this study between December 2020 and April 2021, but Aichi Prefecture, where NCGG is located, was under a state of emergency from mid-January to February 2021; participants responded to our study questionnaire in different infection statuses, and our analyses did not reflect these differences. In addition, we did not include a control group in the present study, and sampling was not random; therefore, our results have limited generalizability to broader populations. To our advantage, however, we were able to collect data on many older adults with MCI in a short period of time because we recruited from individuals who were already participating in the ongoing J-MINT study. In an additional limitation, we conducted a cross-sectional study, and thus, we could not measure changes in psychological resilience over the course of the pandemic; future study needs to conduct longitudinal investigations of changes in psychological resilience over the course of and following the COVID-19 pandemic.

The World Health Organization declared COVID-19 a pandemic in March 2020, and it is ongoing as of this writing. Because it shows signs that its abatement will be slow, it is and will be necessary for involved stakeholders to attempt to minimize long-range impacts on populations affected. This study suggested particularly impact of psychological resilience on older adults with MCI. We found correlations in this study between sleep quality, depressive symptoms, older age, and psychological resilience, and we expect these findings to be useful in developing interventions to provide ongoing support to older adults with MCI who are at risk of poor mental health outcomes. We also recommend continuous assessment of these older adults to help them maintain optimal sleep quality and minimize their depressive symptoms during COVID-19 pandemic restrictions.

## Data Availability Statement

The original contributions presented in the study are included in the article/supplementary material, further inquiries can be directed to the corresponding author/s.

## Ethics Statement

The studies involving human participants were reviewed and approved by the NCGG Ethics Committee. The patients/participants provided their written informed consent to participate in this study.

## Author Contributions

NM and TSu designed the study. NM performed statistical analyses and wrote first draft. NM, TSu, YKu, KU, YKi, HA, and TSa contributed to the interpretation and discussion of results and reviewed the manuscript. All authors contributed to the article and approved the submitted version.

## Funding

This study was funded by a Hori Sciences and Arts Foundation and Longevity Sciences Grant (Grant Number 22-23) from the NCGG. The funders had no role in the preparation of this manuscript.

## Conflict of Interest

The authors declare that the research was conducted in the absence of any commercial or financial relationships that could be construed as a potential conflict of interest.

## Publisher's Note

All claims expressed in this article are solely those of the authors and do not necessarily represent those of their affiliated organizations, or those of the publisher, the editors and the reviewers. Any product that may be evaluated in this article, or claim that may be made by its manufacturer, is not guaranteed or endorsed by the publisher.
